# Splenosis Mimicking Lymphoma Relapse Confirmed by ^18^F-FDG PET/CT and Tc-99m Nano-colloid Scintigraphy Thirty Years After Splenectomy for Trauma

**DOI:** 10.4274/mirt.galenos.2018.44227

**Published:** 2019-03-19

**Authors:** Zehra Pınar Koç, Pelin Özcan Kara, Anıl Tombak

**Affiliations:** 1 Mersin University Faculty of Medicine, Department of Nuclear Medicine, Mersin, Turkey; 2 Mersin University Faculty of Medicine, Department of Hematology, Mersin, Turkey

**Keywords:** Splenosis, nano-colloid, FDG, PET, lymphoma

## Abstract

Splenosis is implantation of the splenic tissue in the abdominal region or elsewhere in the body as a consequence of trauma or splenectomy, which might mimic intra-abdominal involvement of several malignancies. This case report presents a patient with abdominal implants without ^18^F-FDG accumulation confirmed to be splenosis by Tc-99m nano-coloid scintigraphy.

## Abstract (tr)

Splenozis bazı durumlarda malign hastalıkların karın içi tutulumunu taklit edebilen splenik dokunun travma veya splenektomi sonucu karın içi veya başka bölgelere implantasyonudur. Bu olgu sunumunda abdominal implantları olan ve ^18^F-FDG tutulumu göstermeyip Tc-99m nanokolloid sintigrafisi ile splenozis tanısı doğrulanan hastayı sunmak istedik.

## Figures and Tables

**Figure 1:**
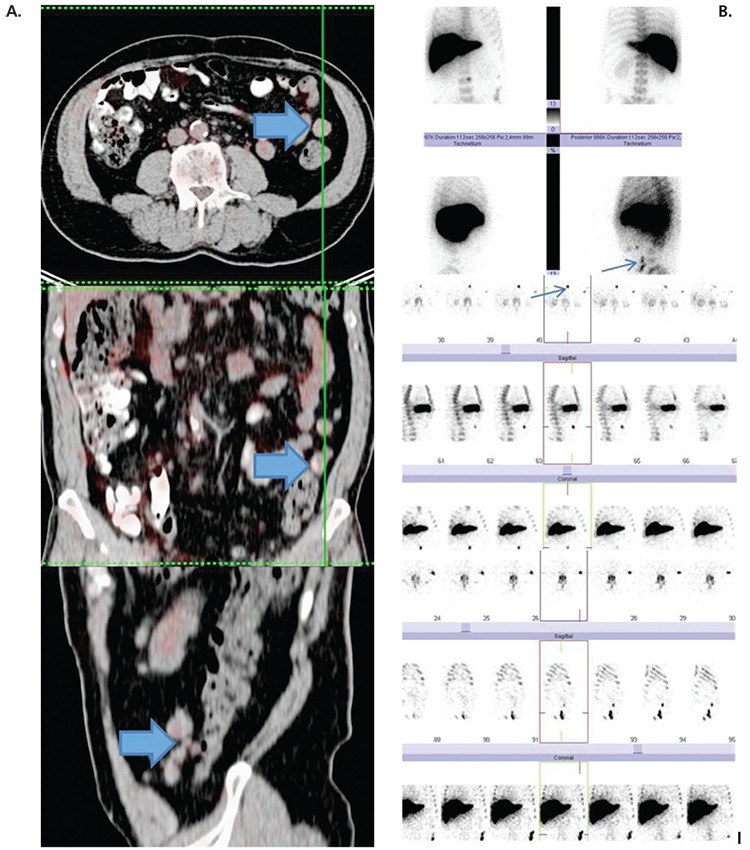
A) Upper to lower; trans-axial, horizontal and sagittal PET/CT fusion images showing faint activity accumulation in intra-abdominal lesions (wide arrows). B) Upper to lower; Tc-99m nano-colloid planar antero-posterior and lateral, SPECT trans-axial, coronal and sagittal projection images of two lesions pointed by narrow arrows. A 64 year-old male patient with a history of chemotherapy for Non-Hodgkin lymphoma had undergone splenectomy following an abdominal trauma due to a traffic accident thirty-years ago and has had an eventless follow-up since then. The patient was also positive for hepatitis C virus. His followup abdominal contrast-enhanced CT showed abdominal implants. The patient was referred for Tc-99m nano-colloid scintigraphy and ^18^F-FDG PET/CT at the same time with suspicion of splenosis and relapse. Sequential Tc-99m nano-colloid scintigraphy, SPECT and ^18^F-FDG PET/CT showed an intraabdominal lesion at midline and multiple lesions in the left lateral area (Figure 1). Due to the increased activity accumulation at spleen scintigraphy and relatively low metabolic activity in the PET/CT the patient was diagnosed as splenosis. Although previous studies have shown that splenosis is not characterized by significantly high FDG activity accumulation (1,2), there are exceptions (3). Recent imaging modalities in the field of nuclear medicine include Ga-68 based imaging that exhibits significantly high splenic uptake which might not differentiate tumor involvement from ectopic splenic tissue, thus requiring further scintigraphic imaging and attention to this particular issue. In a recent case report, peritoneal metastasis was ruled out by selective spleen SPECT/CT imaging in a patient who showed significant intra-abdominal Ga-68 PSMA uptake (4). Although in general splenosis presents with disseminated abdominal lesions, various sites of occurrence have been reported as case reports (5). In a previous case report, hepatic involvement as shown by selective spleen scintigraphy was described (6). Another case report of hepatic splenosis was identified by PET and diagnosed by histopathologic examination (7). False positive somatostatin imaging of a solitary pulmonary nodule and intrathoracic splenosis has also been reported (8). Although the presentation of the patient presented herein was not an unusual manifestation, splenosis is generally not expected after such a long period. To the best of our knowledge, with a diagnosis of splenosis 30 years after splenectomy, this is the longest interval reported in the literature.
